# Advances and applications of gut organoids: modeling intestinal diseases and therapeutic development

**DOI:** 10.1093/lifemedi/lnaf012

**Published:** 2025-03-07

**Authors:** Xiaoting Xu, Yuping Zhang, Guoxin Huang, Ansu Perekatt, Yan Wang, Lei Chen

**Affiliations:** School of Life Science and Technology, Key Laboratory of Developmental Genes and Human Disease, Southeast University, Nanjing 210031, China; School of Life Science and Technology, Key Laboratory of Developmental Genes and Human Disease, Southeast University, Nanjing 210031, China; Clinical Research Center, Shantou Key Laboratory of Basic and Translational Research of Malignant Tumor, Shantou Central Hospital, Shantou 515041, China; Department of Chemistry and Chemical Biology, Stevens Institute of Technology, Hoboken, NJ 07030, United States; Center for Translation Medicine Research and Development, Shenzhen Institutes of Advanced Technology, Chinese Academy of Sciences, Shenzhen 518055, China; School of Life Science and Technology, Key Laboratory of Developmental Genes and Human Disease, Southeast University, Nanjing 210031, China; Institute of Microphysiological Systems, Southeast University, Nanjing 211189, China

**Keywords:** organoid, intestinal disease, personalized medicine, therapeutic strategy, microenvironment

## Abstract

Gut organoids are 3D cellular structures derived from adult or pluripotent stem cells, capable of closely replicating the physiological properties of the gut. These organoids serve as powerful tools for studying gut development and modeling the pathogenesis of intestinal diseases. This review provides an in-depth exploration of technological advancements and applications of gut organoids, with a focus on their construction methods. Additionally, the potential applications of gut organoids in disease modeling, microenvironmental simulation, and personalized medicine are summarized. This review aims to offer perspectives and directions for understanding the mechanisms of intestinal health and disease as well as for developing innovative therapeutic strategies.

## Introduction

Intestinal epithelial cells (IECs) are among the most rapidly renewing tissues in adult mammals, with a renewal cycle of every 3 to 5 days [1]. The intestinal epithelium is structured into crypts (invaginated regions) and villi (projecting regions), with intestinal stem cells (ISCs) residing at the base of the crypts. The primary differentiated epithelial cell types include enterocytes, which are the most abundant and play an essential role for nutrient absorption, along with Paneth cells, goblet cells, enteroendocrine cells (EECs), and tuft cells [2–4]. Notably, Paneth cells are specific to the small intestine and are absent in the colon and rectum. IECs act as a physiological barrier, playing crucial roles in nutrient absorption and metabolism, immune regulation, and mediating interactions between host and microbial communities. The remarkable self-renewal capacity of IECs, combined with their diverse cell types and specialized functions, makes them a vital biological resource for studying intestinal diseases [5].

Organoid technology is a new type of tissue engineering technology that leverages the self-organizing ability of stem cells to grow in a three-dimensional culture environment [6–8]. Scientists have a long history of exploring *in vitro* culture techniques to simulate human and animal organs [9]. In 1907, Henry Van Peters Wilson conducted the first documented attempt at *in vitro* organism regeneration. He demonstrated that dissociated sponge cells have the remarkable ability to self-organize and regenerate into a complete organism [10]. The modern use of “organoid” gained prominence with advancements in stem cell and three-dimensional (3D) culture technologies. These organoids could replicate key structural and functional aspects of various organs. In 2007, Hans Clevers and his team, utilizing cell lineage tracing techniques, made a groundbreaking discovery by Lgr5^+^ intestinal stem cells (ISCs) at the base of intestinal crypts, demonstrating their capacity for self-renewal [11]. Building on this discovery, in 2009, Sato et al. reported the use of adult intestinal stem cells to form 3D intestinal organoids in a 3D matrix [12]. Unlike traditional primary cell cultures or established cell lines, organoids offer a significant advantage in their cellular complexity. They closely replicate the diverse cellular architecture of tissues from experimental animals or humans, preserving essential physiological properties of *in vivo* organs.

By exploring disease mechanisms and evaluating therapeutic efficacy, organoid technology can effectively reduce failure rates during the clinical development of new drugs [13, 14]. Organoid technology not only provides an *in vitro* model for partially mimicking disease states and microenvironments, providing an *in vitro* model that closely replicates *in vivo* conditions for studying disease mechanisms and developing novel therapeutic approaches, but has also demonstrated potential in drug screening and organoid transplantation, offering new strategies for disease treatment. In our previous studies, we employed an *in vitro* intestinal organoid model to identify the critical functions of HNF4 transcription factors in intestinal development. Our findings revealed their regulatory interactions with the BMP/SMAD signaling pathway and highlighted their importance in metabolic regulation [15, 16]. Furthermore, we discovered that transforming growth factor β1 (TGFB1) significantly enhance the transplantation potential of intestinal organoids by inducing fetal-like gene expression, which facilitates their adaptation and survival in damaged intestines in animal models [17]. This review provides an overview of intestinal organoid cultivation techniques and their applications in simulating the intestinal microenvironment, constructing disease models, screening drugs, and exploring organoid transplantation, providing a theoretical foundation and guidance for the broader application of intestinal organoids.

## Cultivation of intestinal organoids

**Figure 1.**
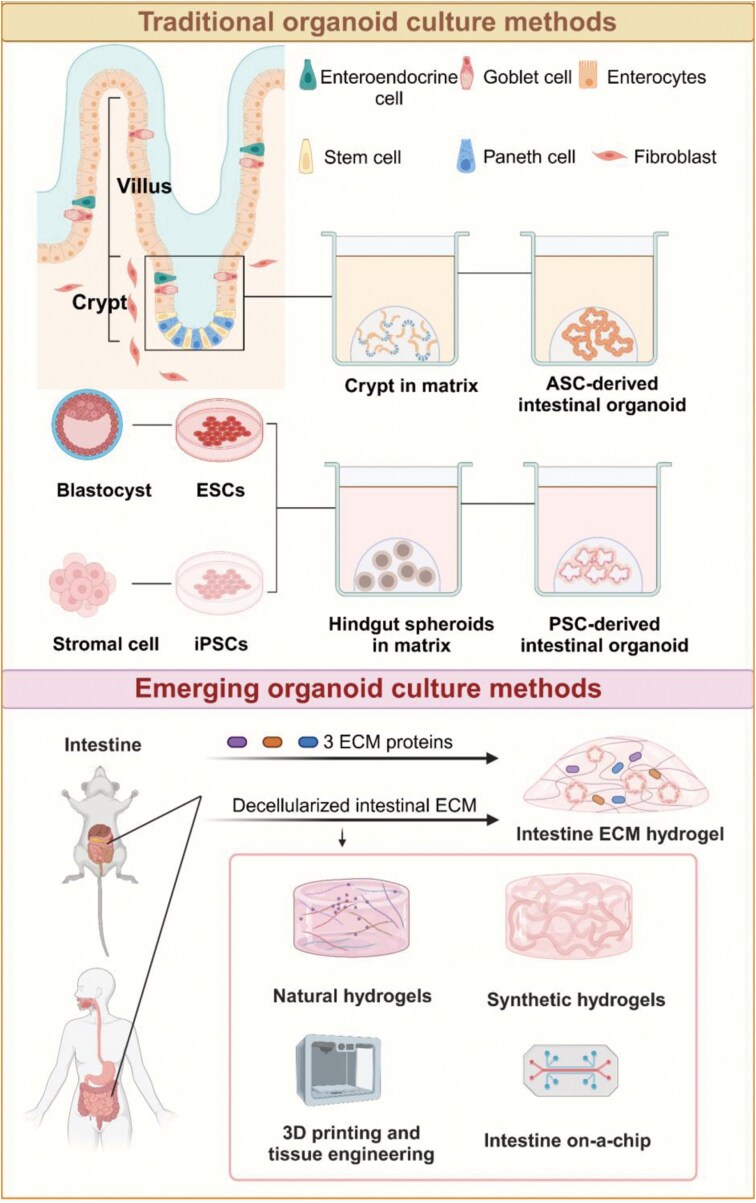
**Approaches for intestinal organoid cultivation.**The top panel illustrates traditional methods for cultivating intestinal organoids, categorized into two approaches: 1) Isolating intestinal crypts and embedding them in Matrigel to generate organoids derived from ASCs; 2) Deriving stem cells from ESCs or iPSCs and culturing them in Matrigel to generate intestinal spheroids. The bottom panel presents emerging methods for organoid cultivation, including the use of intestinal extracellular matrix (ECM) hydrogels generated from decellularized intestinal tissue, artificial and tunable synthetic hydrogels, 3D printing technology to fabricate hydrogels with complex tissue structures, and microfluidic systems that replicate the dynamic microenvironment of the intestine.

Intestinal organoids can be generated from adult stem cells (ASCs) and pluripotent stem cells (PSCs), including embryonic stem cells (ESCs) and induced pluripotent stem cells (iPSCs). Traditional culture techniques rely heavily on Matrigel, which provides structural support and certain growth factors to partially mimic the *in vivo* microenvironment, supporting the growth of intestinal organoids. Emerging approaches, such as hydrogels, further optimize organoid development by modulating biophysical signals, advancing research in intestinal organoid technology (Fig. 1, top panel).

Matrigel is commonly used in conjunction with key components such as R-spondin1 (which activates the Wnt signaling pathway), epidermal growth factor (EGF, which promotes small intestinal cell proliferation), and Noggin (which inhibits BMP activity). Together, these elements partially replicate the signaling pathways present in stem cell niches *in vivo*, promoting organoid growth and development [18, 19]. To culture colon organoids, Wnt-3a is to compensate for insufficient Wnt signaling in the absence of Paneth cells and other Wnt-producing cells. Human intestinal organoids require supplements, including gastrin, niacinamide, N-acetyl-L-cysteine, a transforming growth factor receptor type I (TGF-β R1) inhibitor (A83-01), and a p38 mitogen-activated kinase (MAPK) inhibitor (SB202190), to support their growth, which differs from that of murine organoids [20].

ASC-derived intestinal organoids are typically cultured from crypts or Lgr5^+^ ISCs. During the early stages of growth, these organoids initially form spherical cysts composed of progenitor cells, which subsequently develop into a 3D structure characterized by a “budding” pattern within 2–4 days [12, 21]. These organoids can be passaged for long-term culture while maintaining genetic stability. PSC-derived organoids, on the other hand, can replicate the process of intestinal development. Spence et al. were the first to successfully establish human intestinal organoids (HIOs) from iPSCs using successive growth factor manipulations that mimic embryonic gut development [22]. The development of PSC-derived organoids occurs in three stages: expansion of iPSCs and differentiation into definitive endoderm (DE), formation of intestinal spheroids through the outgrowth of the DE monolayer, and maturation of these spheroids after embedding them in a stromal gel for 28 days. Unlike ASC-derived organoids, PSC-derived organoids offering a more comprehensive model of intestinal development. These organoids can differentiate into both epithelial and mesenchymal tissues, though they still lack other components such as neural and immune cells [22–25] (Fig. 1, top panel).

Matrigel is widely used as the standard material for cultivating intestinal organoids due to its broad availability. However, its batch-to-batch variability and animal-derived origin present challenges for achieving reproducibility and scalability. Researchers are increasingly exploring hydrogels as a promising alternative. Hydrogels, both natural and synthetic, offer superior biocompatibility and flexibility in modulating the microenvironment. Their 3D network structure mimics stem cell niches, with well-defined, biocompatible compositions that influence organoid development through various biophysical signals [26–28] (Fig. 1, bottom panel).

For example, natural hydrogels could provide more favorable environments for organoid survival compared to synthetic alternatives [29, 30]. The density of alginate polymers has been shown to significantly impact organoid survival by modulating the mechanical properties and nutrient diffusion, whereas cellulose-based media enhance adhesion between organoids and nanofibrillated cellulose, supporting the formation of small intestinal organoids [28, 31]. Additionally, natural polysaccharides, such as FP001 and FP003, facilitate large-scale organoid formation through suspension culture by enhancing cell aggregation and providing a stable suspension environment [32]. CS-GelMA composite hydrogels could potentially promote organoid formation by modulating YAP expression via mechanical regulation [33]. In summary, natural hydrogels not only offer advantages in biocompatibility but also optimize organoid culture conditions through various mechanisms (Fig. 1, bottom panel).

Emerging synthetic hydrogel scaffolds such as polyethylene glycol (PEG) are highly suitable for organoid culture because of their ease of engineering, ability to bind signaling molecules, and customizable properties. For example, embedding mouse intestinal stem cells in PEG hydrogels containing RGD peptides (arginine-glycine-aspartic acid) enhances cell survival and facilitates organoid formation [34]. PEG-4MAL is known for its low protein adsorption, easy incorporation of cell adhesion peptides, and low inflammatory response. It has been used to advance tissue implantation and wound healing research [35] (Fig. 1, bottom panel).

Researchers have been advancing the design and customization of hydrogel scaffolds to facilitate personalized organoid culture systems. For instance, QGel CN99 addresses key limitations associated with Matrigel by enabling the establishment and long-term expansion of intestinal organoids while preserving their stemness and differentiation potential [36]. Studies have demonstrated that different culture media significantly influence organoid growth, stem cell differentiation, and intestinal disease modeling. For instance, Sato et al.’s medium (S-medium) supports stable organoid growth but results in lower expression levels of pharmacokinetic-related genes, making it less suitable for drug metabolism studies. Fujii et al.’s medium (F-medium) promotes greater cell diversity, including secretory cells, but features slower growth and moderate enzyme activity. In contrast, Miyoshi et al.’s medium (M-medium) enhances the expression of pharmacokinetic-related genes (e.g. *CYP3A4*) and enzyme activity, closely mimicking adult human intestinal tissue, making it more appropriate for pharmacokinetic applications [37]. In the context of disease modeling and personalized medicine, tailored media, incorporating niche-specific factors such as Wnt3a, R-spondin1, and Noggin, are essential. These modifications enable the development of patient-derived organoids (PDOs) that replicate tumor heterogeneity and facilitate drug testing for colorectal cancer (CRC) and other intestinal diseases. Additionally, optimized organoid media for PSCs and ASCs support the generation of diverse cell lineages and complex tissue structures, advancing research in regenerative medicine and epithelial function.

Additionally, incorporating biologically derived cells into the medium can elicit specific biological responses [38, 39], such as enhancing vascularization within organoids [40] or more effectively replicating the intestinal microenvironment [41]. Innovations in hydrogel technology, including thermo-responsive [42] and photo-responsive [43] properties, are further broadening the scope for precise and adaptive optimization of intestinal organoid cultures. The integration of advanced manufacturing technologies, such as 3D printing, has further improved the efficiency and reproducibility of organoid research. For example, photopolymerization-based 3D printing with bioactive resin has been demonstrated to closely mimic the physiological and structural characteristics of native tissues [44]. Additionally, organoid biochips have emerged as a valuable tool to replicate the complex microenvironment and functional attributes of human organs *in vitro* [45]. Collectively, these advancements highlight hydrogel-based materials as a promising alternative to Matrigel, offering significant potential for organoid engineering. These materials pave the way for novel research directions and practical applications in the development and study of intestinal organoids (Fig. 1, bottom panel).

## Intestinal organoids and their microenvironment

**Figure 2.**
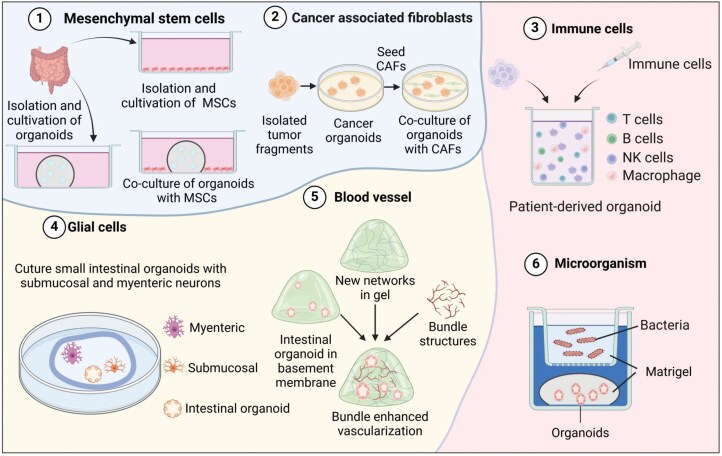
**Simulation of intestinal microenvironment using intestinal organoids.**Various approaches to integrating organoids with different cell types or microorganisms include: 1) MSCs isolated from tissue samples and combined with organoids. 2) Cancer organoids derived from tumor tissues paired with CAFs to study tumor-stromal interactions. 3) Patient-derived organoids integrated with immune cells, including T cells, B cells, NK cells, and macrophages, to mimic immune microenvironments. 4) Small intestinal organoids cultivated alongside submucosal and myenteric neurons from the intestinal nervous system to explore neural interactions. 5) Organoids embedded in hydrogels containing vascular networks to promote vascularization and simulate blood supply. 6) Bacteria or other microorganisms introduced to organoids to model host–microbe interactions.

Intestinal organoids aim to mimic the *in-vivo* organ. However, tissues obtained from biopsies often contain only epithelial cells, which fail to fully capture interactions with other mucosal cell types within organoid cultures [46]. This limitation arises because epithelial stem cells inherently differentiate exclusively into epithelial lineages, thereby excluding other cell types that are critical for representing the full complexity of the intestinal microenvironment. Notable limitations include the absence of a functional vascular system, smooth muscle, Cajal mesenchymal cells, connective tissue containing fibroblasts, and components of the nervous and immune systems. These shortcomings have prompted researchers to explore co-culture methods that incorporate specific cell types to enhance organoid functionality and complexity [47–50]. Efforts have focused on integrating these factors into PSC/ASC-derived epithelial organoids to better replicate *in vivo* conditions, as these cells are integral to organ function and tissue homeostasis [51, 52]. For instance, co-culturing organoids with macrophages has been used to study gemcitabine resistance in cancer models, highlighting the role of immune cells in drug resistance mechanisms [49]. Similarly, co-culture with dendritic cells has provided insights into immune responses in CRC, particularly in how tumor cells interact with immune cells to modulate the tumor microenvironment [53]. Organoids co-cultured with intestinal T-resident memory cells have provided valuable information on the role of lymphocytes in inflammatory diseases such as Crohn’s disease (CD), offering potential avenues for therapeutic intervention [54]. Additionally, including endothelial progenitor cells and organ-specific fibroblasts has been shown to enhance the angiogenic capacity of organoids, facilitating the formation of a more mature vascular-like network [55]. While clinical translation remains a long-term goal, recent advancements in multicellular co-culture systems, combined with innovations in material science and technology, are critical for more accurately replicating the intestinal microenvironment and evaluating therapeutic efficacy in preclinical models (Fig. 2).

### Co-culture with mesenchymal stem cells (MSCs)

MSCs possess strong immunomodulatory abilities, which allow them to influence immune responses and facilitate tissue repair, making them ideal for remodeling the intestinal immune environment and repairing the epithelial barrier. Co-culturing MSCs with intestinal organoids has become a significant approach for studying the mechanisms of intestinal injury repair. Lin et al. developed a culture system that assembles intestinal epithelial cells and MSCs, which more accurately replicated the development and differentiation processes of intestinal crypts compared to traditional intestinal organoid models [56]. Additionally, Yetkin-Arik et al. developed a co-culture model that combined small intestinal human organoids and human bone marrow-derived MSCs to study chemotherapy-induced injury and the regeneration of damaged intestinal epithelium through MSC treatment [57]. Further studies revealed that secreted factors from MSCs could enhance regeneration of intestinal organoids and mouse intestinal tissues by activating the Wnt-Notch signaling pathway [58] (Fig. 2, panel 1).

### Co-culture with cancer-associated fibroblasts (CAFs)

CAFs play a crucial role in the tumor microenvironment by promoting cancer cell proliferation, maintaining stem cell properties, and enhancing drug resistance [59]. Calon et al. employed single-cell mass spectrometry and machine learning methods to analyze the responses of PDOs and CAFs from CRC patients to clinical therapies. Their study highlighted the importance of personalized therapy based on patient-specific drug responses [60, 61]. In 2018, a study demonstrated the successful generation of intestinal organoids from iPSCs by supplementing with Wnt3a and fibroblast growth factor 2 [62]. Luo et al. further developed a co-culture model by encapsulating PDOs in three-dimensional hyaluronic acid-gelatin hydrogels with patient-derived CAFs. This approach successfully mimicked the tumor microenvironment and maintained the molecular characteristics of the original patient tumors [63]. A subsequent study used organoid microarrays to optimize the co-culture conditions between PDOs and CAFs, recreating the intestinal microenvironment. This 3D model has significant potential for evaluating immunotherapeutic drugs [64] (Fig. 2, panel 2).

### Co-culture with immune cells

The adult gastrointestinal tract has a diverse population of immune cells that are essential for maintaining homeostasis. The balance between immune and epithelial cells is crucial, and disruptions can lead to inflammation. Studies have demonstrated that immune cell-derived factors play key roles in maintaining microenvironmental homeostasis when immune cells are co-cultured with intestinal crypts [65–68]. Co-culture systems have been established to study interactions between intestinal organoids and immune cells, including T cells, macrophages, and B cells [69–71]. Recent research has focused on the optimization of these co-culture systems. For example, Neal et al. developed a gas–liquid interface co-culture system, embedding tumor organoids and immune cells in a collagen matrix [72]. Múnera et al. successfully generated functional macrophages from blood-derived endothelial-like cells and erythroid progenitor cells [73]. Co-culturing mouse-derived intestinal cells with syngeneic bone marrow-derived macrophages effectively eliminated potential allogeneic reactions between immune and epithelial cells [74]. Kakni et al. developed a micropore-based co-culture system for mouse intestinal organoids and RAW 264.7 macrophages. This system enables continuous monitoring of cell interactions and supports high-throughput downstream applications [75]. Beyond the *in vitro* co-culture system, *in vivo* studies have leveraged the renal capsule as a transplantation site for organoids, offering new avenues for co-culture research with immune cells [76] (Fig. 2, panel 3).

### Co-culture with glial cells

The enteric nervous system (ENS) regulates intestinal functions, including digestion and immunity, through a network of neurons and glial cells within the intestinal wall [77]. Enteric glial cells are widely distributed across the gut and are closely connected to the ENS and nerve fibers that innervate the intestine. In 2017, Workman et al. pioneered the co-culture of neural crest cells (NCCs) with intestinal organoids, demonstrating that NCCs could differentiate into both neurons and glial cells [78]. Llorente et al. employed microinjection techniques to introduce fluorescently labeled compounds into the organoid lumen and established an innervation system for intestinal organoids by co-culturing myenteric and submucosal neurons with intestinal organoids [79]. The ability to mimic the gut microenvironment more accurately is crucial for exploring the role of nerves within the gut (Fig. 2, panel 4).

### Vascularized epithelial organoids

The vascular endothelium is vital for intestinal barrier, blood supply, nutrient transport, and immune cell migration. However, the absence of vascularization in organoid cultures has hindered their ability to fully replicate the intestinal microenvironment. Vascularization has traditionally been achieved through *in vivo* models, where organoids could be transplanted into highly vascularized regions of immunocompromised mice to achieve extensive vascularization [80]. Recent efforts have focused on *in vitro* approaches using microfluidics [81, 82] and organoid microarrays [55, 83]. Endothelial cells capable of secreting vascular growth factors are also being explored to induce vascularization of organoids *in vitro* [84]. A recent study introduces an innovative culture system that supports vascular development, organoid expansion, and vascularization of primary cells by incorporating basic fibroblast growth factor (bFGF) and heparin [85]. These innovations are laying the groundwork for a more comprehensive understanding of the intestinal microenvironments (Fig. 2, panel 5).

### Co-culture with microorganisms

The study of gut microbes and their interactions with the host requires models that closely replicate key features of the gut epithelium, such as a polarized epithelial layer consisting of diverse cell types, along with the capacity to mimic the absorptive, immune, and neuroendocrine functions of the human gut. Disruptions in these microbial interactions lead to ecological dysregulation, which may impair the epithelial barrier, weaken mucosal defenses, and activate immune cells. Intestinal organoids have become essential tools for studying these interactions. However, the 3D structure of traditional organoid culture models presents challenges in accessing the inner cells, particularly those on the luminal surface facing the organoid lumen, which is critical for direct interactions with microorganisms. To overcome this, several strategies have been developed to enhance the co-culture of microbes with intestinal organoids, including microinjection [86–88], suspension culture with intestinal organoids [89, 90], organoid-derived monolayers [91, 92], and organoids with reversed polarity [93] (Fig. 2, panel 6).

The microinjection method involves injecting microorganisms directly into the lumen of organoids, facilitating interactions between the microbes and the apical surface of the organoid cells. This technique is precise, efficient, and minimally toxic to the organoid cells. However, microinjection is technically demanding, requiring advanced equipment and skilled operators, and it may not be feasible for high-throughput experiments. Horvath et al. developed an animal model by first culturing microorganisms in a specific medium, microinjecting them into intestinal organoids, and subsequently transplanting the organoids *in vivo* [94]. Similarly, Zhang et al. employed this technique to elucidate the pathophysiology of *Salmonella* infections in the gut [95].

Organoid-derived monolayers provide another approach by incubating single cells or organoid fragments in media containing microorganisms [96]. These monolayer cultures are particularly effective for co-culturing with aerobic bacteria due to their improved gas exchange and reduced oxygen concentration gradients compared with other systems. However, maintaining anaerobic conditions in such co-cultures requires a continuous supply of nutrients and effective oxygen barriers to minimize oxygen diffusion, especially when dealing with strict anaerobes. To overcome the differing oxygen needs of epithelial cells and microorganisms in the trans-well co-culture system, the Intestinal Hemi-Anaerobic Co-Culture System (IHACS) was developed. IHACS features an anoxic apical chamber and a normoxic basolateral chamber, creating a more suitable environment for human colonic epithelium [97, 98]. This dual-chamber design effectively mimics the oxygen gradients found in the gut while supporting the growth of anaerobic microorganisms and the survival of epithelial cells, thus providing a more physiologically relevant model [99].

Despite these advancements, the morphology of traditional organoids continues to pose challenges for microbial access to the organoid interior. To address this, Co et al. introduced an innovative method of culturing organoids with reversed polarity, where the lumen and basal structures are inverted while preserving the integrity and functionality of the epithelial barrier through optimized extracellular matrix composition and fine-tuned culture conditions that regulate key signaling pathways, such as Wnt and Notch [93]. Although apical-outward organoids face challenges such as reduced proliferation rates and difficulties in controlling differentiation due to altered exposure to growth factors and mechanical cues, they enable direct observation of microbial interactions with the organoid’s interior [100–102]. For example, Dinteren et al. examined the effects of glabridin on the intestinal epithelium of enteroids by leveraging the apical-outward orientation for more direct functional assays [103], while Kakni et al. developed the first apical-outward model of human small intestinal organoids in a hypoxic environment, which better simulates the low-oxygen conditions of the gut lumen, thereby facilitating direct microbial interactions [104]. Microorganisms often trigger immune responses and repair processes, where immune and stromal cells play critical roles. To more comprehensively mimic *in vivo* conditions, studies [75, 105–108] have incorporated immune cells (such as macrophages, dendritic cells, and T cells), stromal components (such as fibroblasts and glial cells), and microorganisms into organoid systems. However, replicating the dynamic and reciprocal signaling between these cells and organoids *in vitro* remains a challenge.

Optimizing culture conditions, particularly for anaerobic gut flora, continues to be a complex and unresolved issue. Oxygen availability tightly regulates bacterial behavior, and its interplay with other gradients, such as short-chain fatty acids and hydrogen sulfide, further complicates the simulation of a realistic gut environment. Advanced technologies, such as microfluidic systems, can create appropriate oxygen gradients to more accurately replicate the complex *in vivo* environment. Despite their promise, these systems face hurdles such as high costs, technical complexity, and limited scalability, which hinder their widespread adoption. Continuous advancements in microbial-gut organoid co-culture systems will allow for a deeper understanding of the role of gut dysbiosis in the progression of intestinal diseases, including inflammatory bowel disease, irritable bowel syndrome, and colorectal cancer, by elucidating disease-specific microbial and host mechanisms (Fig. 2).

## Intestinal organoid simulation for disease models

**Figure 3.**
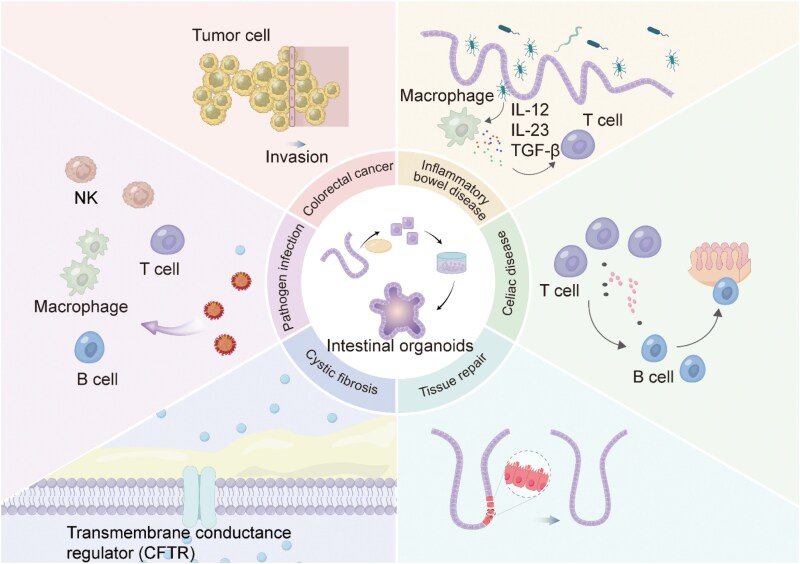
**Simulation of intestinal diseases using intestinal organoids.**1) CRC: Co-culturing CRC patient-derived organoids with CAFs demonstrated that inflammatory CAFs promote EMT. 2) Pathogen infection: Co-culturing intestinal organoids with microbes enabled the investigation of pathogen infections. 3) Cystic fibrosis: Application of CRISPR technology in organoids to study the role of the *CFTR* gene in cystic fibrosis. 4) Tissue repair: High-resolution phenotypic screening platforms based on intestinal organoids have been established to explore the dynamic processes of intestinal injury repair. 5) CeD: The use of intestinal organoids cultured from biopsy samples to study the pathogenesis of celiac disease. 6) IBD: Co-culturing organoids with gut-resident memory T cells provides insights into the role of immune cells in the development of IBD. Furthermore, intestinal organoids cultured from biopsy samples serve as a valuable tool for studying the mechanisms underlying celiac disease.

Simulating the intestinal microenvironment using intestinal organoids aims to recreate the normal physiological state of the gut, incorporating the roles of various cell types, cell–cell interactions, cell–matrix interactions, as well as the influence of microbial communities. A more comprehensive simulation of the intestinal microenvironment allows for a deeper understanding of gut function in healthy states, which is essential for studying intestinal diseases. Disease models built upon this foundation could help explain how pathological changes disrupt these processes. Organoids are capable of replicating the pathological alterations observed in diseases, such as cancer, inflammation, tissue injury, and immune responses (Fig. 3).

### CRC models

CRC develops primarily through two primary pathways: chromosomal instability and microsatellite instability. Given that different CRC subtypes respond differently to specific drugs, individualized treatments are crucial for effective therapy [109]. Tumor organoids retain the histological and genetic mutation characteristics of the original tumor. These organoids have become invaluable preclinical tools, as they preserve tumor heterogeneity and predict individual drug responses. A biobank of CRC organoids from patients enrolled in clinical trials has demonstrated consistency between organoid phenotypes and genotypes, allowing researchers to track tumor changes during treatment and identify vulnerabilities through molecular analysis and drug screening [110]. Research using organoids has provided insight into factors that influence CRC progression and metastasis [111–115]. Notably, UBXN2A was identified as a novel tumor suppressor in CRC, with higher expression levels correlating with increased survival rates [116]. Additionally, the lncRNA POU6F2-AS1 was found to regulate fatty acids in CRC, promoting cancer cell proliferation [117]. The transcription factor SOX17 has been implicated in coordinating immune responses during early CRC development [118], while the JAK/STAT3 pathway has emerged as a potential therapeutic target [119]. Co-culturing of CRC patient-derived organoids with CAFs revealed that inflammatory CAFs promote epithelial–mesenchymal transition (EMT), whereas myofibroblasts can reverse this effect [120]. These findings underscore the utility of CRC organoids in studying cancer initiation, invasion, and metastasis (Fig. 3, top-left corner).

### Inflammatory bowel disease (IBD) models

IBD results from a complex interplay between genetic factors, immune responses, microbial imbalances, and environmental influences. In IBD patients, disruption of the gut microbiota and damage to the epithelial barrier allow both commensal and opportunistic bacteria to invade intestinal epithelial cells, leading to abnormal immune responses and chronic inflammation [121]. Intestinal organoids have become indispensable for understanding IBD pathogenesis by exploring the relationships between genetic susceptibility, environmental factors, and epigenetic regulation. For instance, CRISPR-mediated gene editing in IBD organoids has been utilized to investigate how specific variants, such as those in NOD2, impact cellular functions and barrier integrity [75, 122]. Susceptibility gene variants in IBD can affect cellular junction assembly, increasing IECs permeability and enhancing interactions between antigens, microbes, and the immune system [123].

Co-culturing IBD organoids with inflammation-associated fibroblasts (IAFs) showed that IAFs activate cystic fibrosis transmembrane conductance regulators (CFTR) via prostaglandin E (PGE)-mediated signaling pathways, disrupting ion balance and contributing to epithelial damage [124]. Studies have also mapped the transcriptional landscape of IBD organoids in response to cytokines mediating mucosal immune responses [125]. Pro-inflammatory cytokines, such as IL-20, IL-6, IL-1β, and IL-17, have been implicated in impairing epithelial barrier function [126–130]. Hammoudi et al. demonstrated that co-culturing IBD organoids with intestinal T-resident memory cells, along with inhibiting CD103 and NKG2D, can block lymphoepithelial interactions, thereby reducing cytotoxic immune responses and alleviating tissue damage associated with IBD symptoms [54].

The gut microbiota and its metabolites, which play crucial immunomodulatory and metabolic roles, undergo significant alterations in IBD patients. Notably, changes in short-chain fatty acids [131–133], bile acid derivatives [134–136] and tryptophan metabolites [137, 138] have been observed. Giri et al. discovered that certain *Clostridium* strains could inhibit immune-mediated NF-κB activation in intestinal organoids [139]. Colonic organoid models have significantly improved the clinical translation of IBD therapeutics. For instance, Zein/SA/BG shows promise in treating IBD by modulating macrophage polarization and promoting tissue regeneration [140]. Additionally, Kim et al. found that Schisandrin C enhances epithelial barrier formation by upregulating ZO-1 and occludin in both intestinal cell monolayers and organoids [141]. IBD organoid models offer a valuable platform for individualized disease modeling, preserving the morphological structure and genetic characteristics of the diseases (Fig. 3, top-right corner).

### Tissue repair

Intestinal epithelial cells have a remarkable ability to self-repair after injury. Organoids, which mimic the structure and function of physiological epithelial tissues, have emerged as powerful tools for studying mechanisms of epithelial repair. Upon injury, the number of Lgr5^+^ crypt basal columnar cells decrease, whereas slow-cycling reserve stem cells resistant to DNA damage are activated to facilitate repair. Researchers developed a novel intestinal organoid culture system, which is enriched with multiple regenerative stem cell populations, including injury-responsive *Lgr5*^+^ and *Clu*^+^ cells [142]. Lukonin et al. constructed a phenotypic screening platform based on single-cell culture of intestinal organoids, enabling high-resolution analysis of individual cell behaviors and phenotypes during regeneration, including proliferation, differentiation, and signaling dynamics [143]. Our previous study has shown that TGFB1 promotes a regenerative state in intestinal organoids that resembles fetal-like regeneration by activating the YAP–SOX9 signaling pathway in the epithelium [17]. In regenerative medicine, implanting organoids into the host has been shown to facilitate maturation into functional gut tissues. For example, mouse colonic organoids embedded in type I collagen gel and transplanted into damaged colonic regions effectively treated ulcerative colitis in a mouse model [144] (Fig. 3, bottom-right corner).

### *Cystic fibrosis (*CF*) models*

CF is a severe autosomal recessive disorder that significantly affects the life expectancy of patients [145]. *In vitro* drug response in CF patient-derived organoids closely correlates with clinical outcomes. CF primarily results from mutations in the *CFTR* gene. To address this, researchers investigated the role of CFTR in the maturation of the human intestine using patient-derived intestinal samples *in vitro* and HIOs implanted *in vivo*. CFTR levels were quantified using a forskolin-induced swelling assay [146, 147]. Recent advances include the use of CRISPR-based prime editing to correct *CFTR* mutations, leading to significant restoration of CFTR function [148] (Fig. 3, bottom-left corner).

### Pathogens infection models

HIOs have proven to be valuable models for understanding rotavirus infection, supporting viral replication and generating infectious viral particles [149–151]. In addition to viral infections, organoids have also been used to study the effects of parasitic infections. For example, *Giardia* infection in intestinal organoids has been shown to alter ion channels and tight junction proteins, leading to barrier dysfunction through adenylate cyclase signaling [152] (Fig. 3, left middle).

### Celiac disease (CeD) models

CeD is an immune-mediated disorder primarily triggered by dietary gluten. Gluten-derived peptides bind to major histocompatibility complex (MHC) class II human leukocyte antigen molecules, HLA-DQ2 or HLA-DQ8, resulting in duodenal mucosal damage [153–155]. Dotsenko et al. provided a detailed process for culturing intestinal organoids from biopsy and developed protocols for detecting viral infection and transglutaminase activity [156]. This method for assessing gluten’s immunogenic potential offers new avenues for CeD treatment [157]. Further research by Verdu et al. revealed significant differences in phenotype and gene expression between organoids derived from crypt stem cells of healthy individuals and those from CeD patients. These findings may help explain the abnormalities in crypt and villus development observed in CeD [158], and a deeper understanding of these differences could offer critical insights into disease mechanisms. Additionally, Conte et al., in their investigation of CeD mechanisms, found that prolamins activate the mTOR/autophagy pathway and induce an inflammatory response in the intestinal organoids of CeD patients, while *Lactobacillus paracasei* was shown to mitigate these harmful effects [159]. This suggests that HLA-restricted, prolamin-specific intestinal T-cell responses are central to CeD pathogenesis [160]. A recent study identified interleukin-7 (IL-7), produced by T cells, as a gluten-induced pathogenic regulator that modulates NKG2C/D expression in CD8 T cells [161]. This innovative CeD organoid model, which incorporates multiple epithelial, stromal, and immune cell types, successfully simulates gluten-induced intestinal epithelial damage and the progression of CeD (Fig. 3, right middle).

## Drug screening

Each commonly used model system for drug screening has its limitations. Two-dimensional cell lines are simple and inexpensive to culture, but they fail to capture the complex structure, behavior, and drug responses of natural tissues. Mouse models suffer from species-specific differences that limit their clinical relevance [162]. Patient-derived xenograft (PDX) models are more representative of human cancers but are impractical for large-scale drug screening due to their high cost and time constraints [163, 164]. Organoids are promising models for precision medicine [165–168], accurately mimicking patient-specific responses to treatment. These physiologically relevant, personalized cancer models that are well-suited for drug development and clinical applications [169, 170]. In particular, intestinal organoids show great potential in drug screening, especially for CRC. Since 2015, research has successfully replicated tumor characteristics using PDO models for high-throughput drug screening. Establishing a bio-screening library in advance could further accelerate drug discovery. Luo et al. developed a high-risk colorectal adenoma (HRCA) organoid model that was subjected to high-quality HRCA drug screening [171]. These PDOs closely mirrored the genetic characteristics of primary tumors, including gene expression, proliferation, and drug responses. Cartry et al. tested 25 PDOs models, identifying effective anticancer drugs with 75% sensitivity and specificity in predicting clinical drug responses [172]. Similarly, Mao et al. tested 335 drugs, identifying 34 drugs with anti-CRC effects [173].

Organoid technology has also been used to screen various aspects of energy metabolism [174], pharmacokinetics [175], drug toxicity [176], and bioavailability [177, 178]. For example, Zheng et al. employed organoids as drug-screening models to identify mitochondria-targeted drugs [179]. The combination of organoid technology with other emerging technologies provides a more realistic platform for drug screening. For example, 3D printing technology allows precise control over the spatial arrangement of cells and biomaterials, enabling the construction of complex tissue structures. Tebon et al. combined bioprinting with high-speed live cell interferometry, improving the quality of drug screening [180]. Microfluidics, when integrated with organoids, provide continuous medium perfusion and promote sustained organoid growth [181].

**Figure 4.**
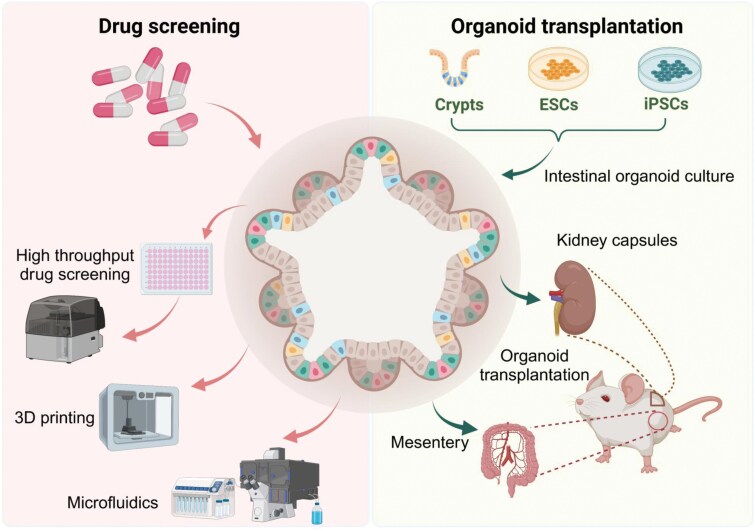
**Drug screening and organoid transplantation.**Left panel: Organoid-based drug screening is conducted using high-throughput screening, 3D printing, and microfluidic technologies. Right panel: Cells derived from crypts, ESCs, or iPSCs are cultured into organoids and transplanted onto the mesentery or under the kidney capsule.

The field of organoid applications is rapidly expanding, with a trend toward more complex and sophisticated model systems. Advancements in organoid culture techniques, such as refining extracellular matrix plates [182], integrating CRISPR-based genetic screening [183], and employing cellular microengineering, are making significant strides. Brandenberg developed microengineered cell culture devices to generate thousands of individual intestinal organoids for suspension cultures and real-time analysis. These devices were used to screen anticancer drug candidates from patient-derived CRC organoids, applying high-content image-based phenotyping to gain insights into drug mechanisms [184]. Additionally, improvements in data analysis after drug screening, such as *Z*-stack imaging to capture the maximum cross-section of organoids in a single session [185] and multifactorial analysis combined with computerized screening to construct and evaluate predictive models [186], enhance the accuracy and efficacy of drug screening (Fig. 4, left panel).

## Organoid transplantation

Although HIOs have demonstrated certain intestinal functions *in vitro*, this model remains inadequate for studying the effects of a wide range of physiological stimuli on human intestinal tissues. Specifically, *in vitro* HIOs lack critical features, such as vascularization, neural innervation, and integrated immune components, all of which are essential for accurately replicating the human intestinal microenvironment. To overcome this limitation, Watson et al. developed an *in vivo* HIO implantation model that successfully produced mature, functional human gut tissue. They embedded HIOs in type I collagen and transplanted them into the kidney capsules of immunocompromised NOD SCID gamma (NSG) mice. This method not only increased HIO size but also significantly enhanced vascularization [187]. In a follow-up study, they expanded their transplantation approach by using the mesentery as the anatomical site, providing a more natural environment similar to intestinal tissue. The mesentery shares a blood supply with the intestine, which is believed to support critical functions, such as peristalsis, immune response, and tissue repair [188].

Transplantation of HIOs into both the renal capsule and mesentery was feasible and successful. Organoids from both sites showed significant maturation, with epithelial and mesenchymal structures developing similarly in both the renal capsule and mesentery. Mesenteric transplantation provided unique advantages, including the development of a dominant lumen within the grafts and a more accurate replication of the intestinal blood supply, both of which supported functions like nutrient absorption and mucosal immune responses. However, mice that received mesenteric transplants exhibited higher mortality than those that received renal capsule transplants. The procedure of renal capsule transplantation was technically easier, making it an ideal model for studying developmental gut biology.

Building on these advances, Singh et al. developed a new HIO system that successfully integrated human immune cells by culturing HIOs in a mouse model with a humanized immune system. These immune cells infiltrated the HIO mucosa, closely mirroring the immune landscape of the developing human gut [76, 189]. Further studies on organoid transplantation in IBD have laid the groundwork for related clinical trials [190–192]. The transplantation of organoids has also shown therapeutic potential, as demonstrated by Zhang et al., who transplanted intestinal organoids into mice with intestinal ischemia-reperfusion injury. This significantly improved survival rates and promoted self-renewal of intestinal stem cells [193].

Our previous studies demonstrated that TGFB1 signaling repairs intestinal damage by activating the pro-regenerative factors YAP/TEAD and SOX9. Moreover, TGFB1 pretreatment enhanced the efficiency of intestinal organoid implantation in damaged mouse colons [17]. Cruz-Acuña et al. synthesized a novel hydrogel that supports stable HIO growth and expansion, which can also be used as an injectable vehicle to deliver HIOs directly to the site of intestinal injury, facilitating HIO implantation and colonic wound repair [194]. However, despite these advancements, current intestinal organoid technology still faces challenges, particularly in achieving the size and functionality required to treat larger intestinal defects, such as those seen in short bowel syndrome (SBS) [195]. To address this, Sugimoto et al. developed an SBS model in rats by performing a total jejunectomy and creating a small intestinalized colon (SIC) by transplanting ileum-derived organoids into the colon at the ileocecal junction. This anatomical relocation allowed the SIC to replicate small intestine structures, including villus and lymphovascular systems. Encouragingly, rats that received ileal organoid transplants had significantly higher survival rates compared to those that received colonic organoids [196]. This approach provides a promising foundation for regenerative medicine and offers novel strategies for treating intestinal diseases (Fig. 4, right panel).

## Summary and prospect

**Figure 5.**
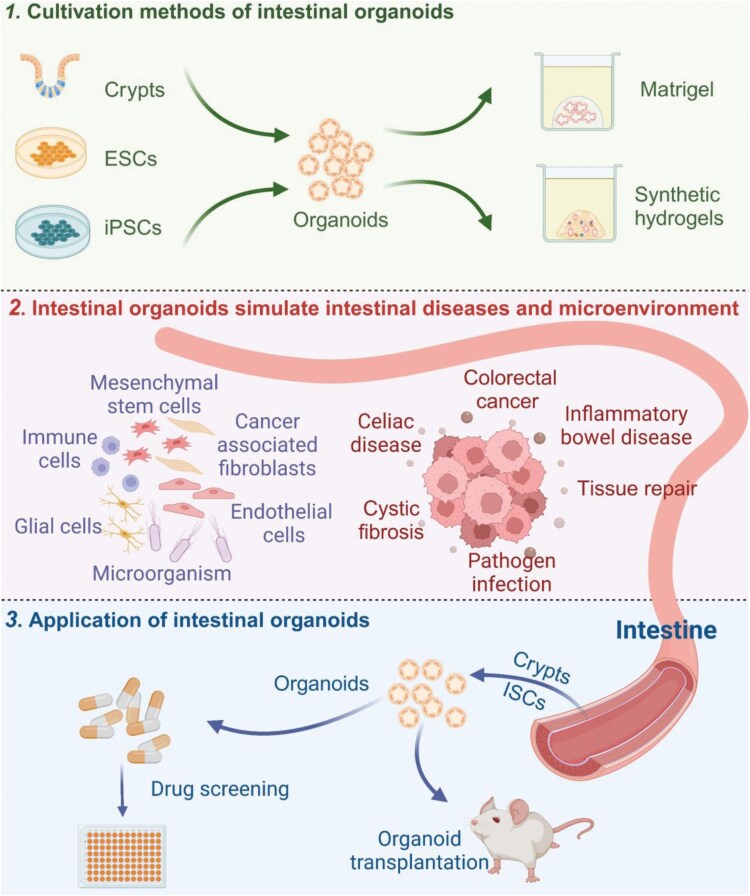
**Approaches and applications of intestinal organoids.**Top panel: Intestinal organoids can be derived from intestinal crypts, ESCs, or iPSCs. They are typically cultured in Matrigel or synthetic hydrogels, which provide structural support for their growth and self-organization. Middle panel: Co-culturing organoids with various cell types, including mesenchymal stem cells, immune cells, glial cells, cancer-associated fibroblasts, endothelial cells, and microorganisms, enables the modeling diverse intestinal diseases. These include colorectal cancer, inflammatory bowel disease, celiac disease, cystic fibrosis, pathogen infections, and tissue repair. Bottom panel: Organoids serve as powerful tools in drug screening and transplantation research, allowing precise evaluation of drug responses and demonstrating significant potential in regenerative medicine.

Organoid technology has made remarkable progress over the past decade, emerging as a powerful tool for modeling intestinal structure and function. This advancement has not only expanded research methodologies in intestinal biology but also opened new avenues for understanding the complex physiological mechanisms of the gut. As research evolves, the application of intestinal organoids in disease modeling, drug screening, and regenerative medicine is becoming more widespread, showing significant potential. Organoids are typically cultivated using stem cells derived from adult or fetal intestinal tissues. These cells differentiate *in vitro* under specific growth factors and matrix environments, forming tissue structures that closely resemble the architecture and function of the gut (Fig. 5, top panel). In recent years, the introduction of microfluidic technology has enhanced the precision of organoid culture, enabling more accurate simulation of the physical and chemical environment of the gut. By optimizing growth factors and matrix compositions, this approach not only improves the physiological relevance of organoids but also provides a more realistic model for drug screening and toxicology studies.

Compared to traditional cell lines and animal models, organoids offer unique advantages in disease research. They more accurately recapitulate the genetic characteristics and microenvironment of the human gut, providing greater credibility in simulating intestinal diseases. Organoids have proven particularly valuable in the study of CRC, IBD, CeD, and SBS. By co-culturing with gut-specific cells, these models can better replicate the mechanisms underlying disease onset. However, current studies often rely on single-cell-type co-cultures, which do not fully capture the complexity of gut physiology. Future research should focus on integrating multiple systems, including immune, neural, and microbial components, into organoid models to develop more comprehensive and realistic gut microenvironments (Fig. 5, middle panel). The advantages of intestinal organoid technology are compelling in drug development, where organoids have demonstrated significant advantages in the early stages of drug screening. Their ability to closely mimic the *in vivo* microenvironment allows for a more accurate reflection of drug bioactivity, toxicity, and metabolism. In particular, organoid-on-a-chip systems, which integrate organoid technology with microfluidics, have improved precision by simulating drug absorption, distribution, metabolism, and excretion processes. This integration of organoid technology with organ-on-a-chip systems has further enhanced the efficiency of screening, reduced the impact of cross-species differences and improved the success rate of clinical translation. As a result, organoid technology has attracted attention from regulatory agencies, emphasizing its potential to replace traditional animal testing in drug development (Fig. 5, bottom panel).

While organoid technology has shown great promise, it is important to address the variability inherent to organoid cultures. Differences in cell sources, culture conditions, and technical procedures contribute to this variability, which poses challenges for reproducibility and scalability. For instance, variations in donor cell genetic background, batch-to-batch differences in matrix materials such as Matrigel, and inconsistencies in manual handling can lead to significant heterogeneity in organoid outcomes. Standardized protocols, robust quality control measures, and high-throughput analysis tools are essential for mitigating these issues. Additionally, the variability in organoid models limits their utility in large-scale drug screening and cross-laboratory comparisons. To address this, it is crucial to develop modular and automated culture systems that minimize human error and ensure uniformity across experiments. Integration of real-time monitoring systems, such as live-cell imaging and biosensors, could further enhance the consistency and reliability of organoid production by providing continuous feedback on growth parameters.

Furthermore, the growing volume of data generated by organoid studies, including imaging, omics, and functional response datasets, requires advanced computational approaches for integration and analysis. These datasets are often complex, multi-dimensional, and heterogeneous, making traditional analysis methods insufficient. The development of AI-powered analytical pipelines and shared organoid databases could enhance data interpretation, promote cross-study comparisons, and accelerate progress in the field. Such databases could include metadata on organoid culture conditions, genetic profiles, and experimental outcomes, providing a centralized resource for researchers to identify patterns and optimize protocols. Collaboration between biologists, bioinformaticians, and engineers will also be critical in addressing these challenges. By leveraging advances in machine learning and cloud computing, the field can establish scalable platforms for data storage, sharing, and analysis, ultimately enabling the full potential of organoid technology to be realized. These efforts will not only improve the reproducibility and scalability of organoid systems but also pave the way for their wider adoption in translational research and precision medicine.

By addressing these challenges and promoting the integration of organoid technology with other cutting-edge techniques such as microfluidics and genome editing, the feasibility of its clinical applications can be significantly enhanced. Organoids possess a unique capability to bridge the gap between simplistic *in vitro* models and complex *in vivo* systems, offering unprecedented opportunities to understand human physiology and disease. With advancements in organoid culture methods, analytical approaches, and their integration into broader research frameworks, the applications of organoids in precision medicine, drug discovery, and regenerative therapies will continue to expand.

The future of organoid research lies not only in overcoming its limitations but also in reimagining the transformative possibilities it brings to science and medicine. Through sustained innovation and collaborative efforts, organoid technology has the potential to revolutionize how we study biology and treat diseases, serving as an increasingly effective tool for exploring intestinal physiology, understanding disease mechanisms, advancing drug development, and driving progress in regenerative medicine.
